# Unexpected outcomes of tislelizumab treatment in thoracic metastasis of malignant phyllodes tumors: a case report and literature review

**DOI:** 10.3389/fonc.2025.1535653

**Published:** 2025-04-14

**Authors:** Yihao Liu, Zhixuan Duan, Minghui Liu, Yongwen Li, Min Wang, Jun Chen, Honglin Zhao

**Affiliations:** ^1^ Department of Lung Cancer Surgery, Tianjin Medical University General Hospital, Tianjin, China; ^2^ Tianjin Key Laboratory of Lung Cancer Metastasis and Tumor Microenvironment, Tianjin Lung Cancer Institute, Tianjin Medical University General Hospital, Tianjin, China

**Keywords:** phyllodes tumors, malignant phyllodes tumors, tislelizumab, immunotherapy, soft tissue sarcoma, FGFR2, KDM6A

## Abstract

Phyllodes tumo (PT) of the breast are classified into benign, borderline, and malignant types. Malignant phyllodes tumor (MPT) with metastasis, particularly those containing sarcomatous components, have a notably poor prognosis. The most common sites of metastasis are the lungs, although metastases can also occur in the pleura and other areas. Metastatic PT is typically treated according to NCCN guidelines for soft tissue sarcomas. The prognosis for patients is extremely poor, with survival typically not exceeding five years. Therefore, the treatment of metastatic MPT presents significant challenges. A 67-year-old female with a history of PT surgery was hospitalized due to acute chest tightness and shortness of breath. MRI revealed a large mass in the left thoracic region, measuring 7.9 × 10.8 × 11.4 cm. A biopsy conducted prior to hospitalization indicated spindle cell soft tissue sarcoma. Due to critical vital signs, she underwent an emergency thoracotomy. Postoperative analysis confirmed the diagnosis of thoracic metastasis from MPT with sarcomatous components. Genetic analysis of the tumor tissue post-surgery revealed a KDM6A gene mutation. Unfortunately, subsequent imaging showed a recurring mass in the left thoracic space, approximately 8 cm in size. Considering the side effects of NCCN-recommended treatments (doxorubicin and ifosfamide) and the high cost of targeted therapies, the patient and her family chose tislelizumab. After six cycles of treatment, the patient’s progression-free survival reached 15 weeks. Due to unsatisfactory treatment effects, the patient and her family decided to discontinue therapy, and the patient passed away in July 2024. Although the combination of surgery and postoperative immune checkpoint inhibitors remains to be validated, this case provides valuable insights into the management of thoracic metastasis from MPT. It offers potential new options for personalized immunotherapy in metastatic MPT.

## Introduction

Phyllodes tumor (PT), also known as cystosarcoma phyllodes, is a rare fibroepithelial tumor composed of stromal and epithelial components. These tumors are classified into three types: benign, borderline, and malignant (1, 2). The first documented case of malignant phyllodes tumor (MPT) with lung metastasis was recorded in 1931, highlighting the possibility of these tumors progressing to malignancy (3). Phyllodes tumors are relatively rare in females. A literature review reports that the overall recurrence rate for PT is approximately 12.6%, with recurrence rates for benign, borderline, and malignant PT at 7.1%, 16.7%, and 25.1%, respectively (4). When distant recurrence occurs, it typically presents as solid nodules or thin-walled cavities in the lungs. Fine Needle Aspiration (FNA) or core needle biopsy is usually insufficient to differentiate PT from fibroadenomas, making surgical excision necessary for an accurate pathological diagnosis (5). Histologically, PT are characterized by their unique leaf-like structures, consisting of dual-layered epithelium with both internal and external myoepithelial components. These tumors may also show pseudoangiomatous stromal hyperplasia and various metaplastic changes, including chondroid, osseous, lipomatous, and stromal giant cell formations. Squamous or apocrine metaplasia within the epithelium is less commonly observed. If features of liposarcoma, osteosarcoma, chondrosarcoma, or rhabdomyosarcoma are present, a diagnosis of MPT can be made (6). Surgical excision with a margin of at least 1 cm is the gold standard for treating local PT. The recurrence rate for benign PT is low, while borderline or malignant PT may have a recurrence rate of 10%-40%. Once MPT metastasizes, the median survival for patients is typically between 5 and 30 months. Adjuvant radiotherapy for MPT has not reached a consensus due to a lack of substantial data; however, it may be beneficial for local recurrence (7). Metastatic or recurrent PT should be treated according to the National Comprehensive Cancer Network (NCCN) guidelines for metastatic soft tissue sarcomas (8). Although immune checkpoint inhibitors (ICIs) have been studied in metastatic soft tissue sarcomas, such studies remain limited, and standard treatment guidelines still primarily recommend chemotherapy (9). Previous studies using doxorubicin alone or in combination with cyclophosphamide showed that only 10-20% of patients with metastatic sarcoma achieved a progression-free survival (PFS) of 4 to 6 months and an overall survival (OS) of 16 to 24 months, with significant side effects (10).

In this case report, we describe a female patient with MPT who experienced recurrence after thoracic surgery and was treated with tislelizumab, achieving a progression-free survival (PFS) of 3.5 months. This case provides new insights into the exploration of immunotherapy and targeted therapy for distant metastasis of MPT.

The ethical aspects of this case report were reviewed and approved by the Ethics Committee of Tianjin Medical University General Hospital. The patient provided written consent for the release of their detailed case information and associated images.

## Case presentation

**Figure 1:**
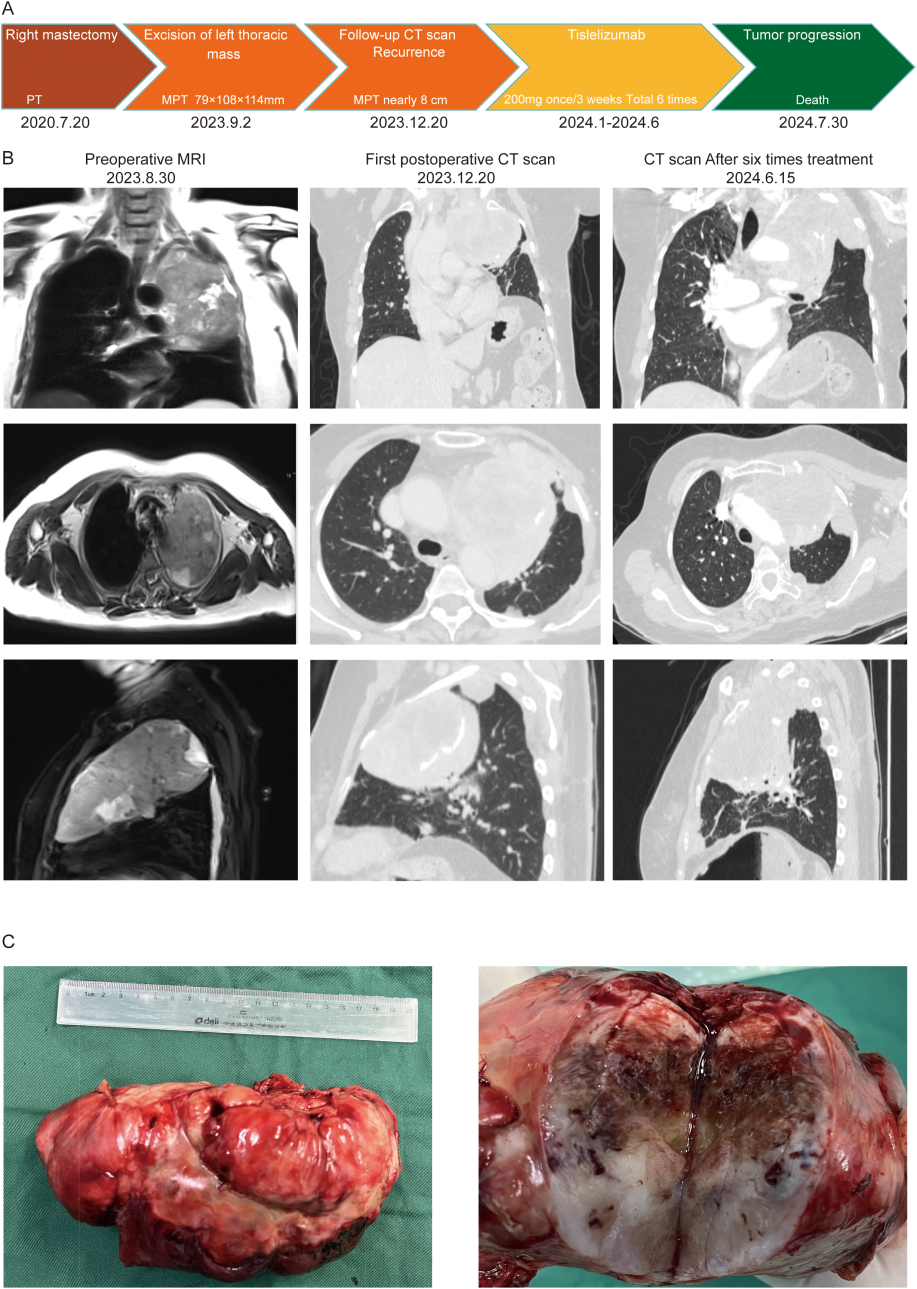
**(A)** Clinical treatment history; **(B)** Imaging data of the patient; **(C)** Tumor size and nature.

On August 30, 2023, a 67-year-old female patient was admitted to the Thoracic Surgery Department of Tianjin Medical University General Hospital due to chest tightness and breathlessness. The complete patient information is presented in **Table 1**, and the entire treatment process is shown in **Figure 1A**. Prior to admission, the patient underwent a CT scan at another hospital, which suggested a lung mass. A thoracic biopsy indicated a spindle cell soft tissue sarcoma, considered low-grade malignant, though a phyllodes tumor could not be excluded. The patient exhibited no symptoms of hemoptysis, fever, or chest pain. Upon admission, the patient underwent a thoracic enhanced MRI, which revealed a large mass in the left chest, approximately 79mm×108mm×114mm, showing uniform enhancement (**Figure 1B**). Prior to the biopsy results, specific laboratory tests, including lung tumor markers (CEA, CYFRA21-A, SCC, and ProGRP), were all negative. The patient had no history of smoking or alcohol consumption, worked in a canteen for many years with exposure to food oil fumes, and had been retired for three years at the time of diagnosis. There was no family history of similar tumors in first-degree relatives. The patient had a history of right mastectomy, and post-operative immunohistochemistry on July 20, 2020, showed positivity for E-cad, CK5/6, and VIM, with a Ki-67 proliferation index of approximately 20%, suggestive of a breast phyllodes tumor (**Supplementary Figure S1A**). Genetic testing revealed an FGFR2 mutation (c.755C>G, p.Ser252Trp, Exon 7; Geno-Truth Dx Lab). The patient had not received chemotherapy or radiation therapy after surgery.

**Table 1:** The patient’s basic characteristics and information.

Characteristics	
Age	67
Gender	Female
Symptoms	Chest tightness and breathlessness
Imaging (MRI)	79mm×108mm×114mm
Smoking history	No
Family history	No
Occupation:	Canteen worker (retired, exposure to cooking fumes from food)
Previous medical history	Breast phyllodes tumors (right mastectomy in 2020) FGFR2 (c.755C>G, p.Ser252Trp, Exon 7)
Pathology	Breast phyllodes sarcoma KDM6A (c. 3510dup, p.Leu1171Serfs*32, Exon24)
Treatment	Tislelizumab (6 cycles,200mg on day 1 every 3 weeks)
Recurrence	Yes
Progression-free survival (PFS)	3.5 months
Overall survival (OS)	11.1 months
Status	Death (2024.7.30)

**Figure 2:**
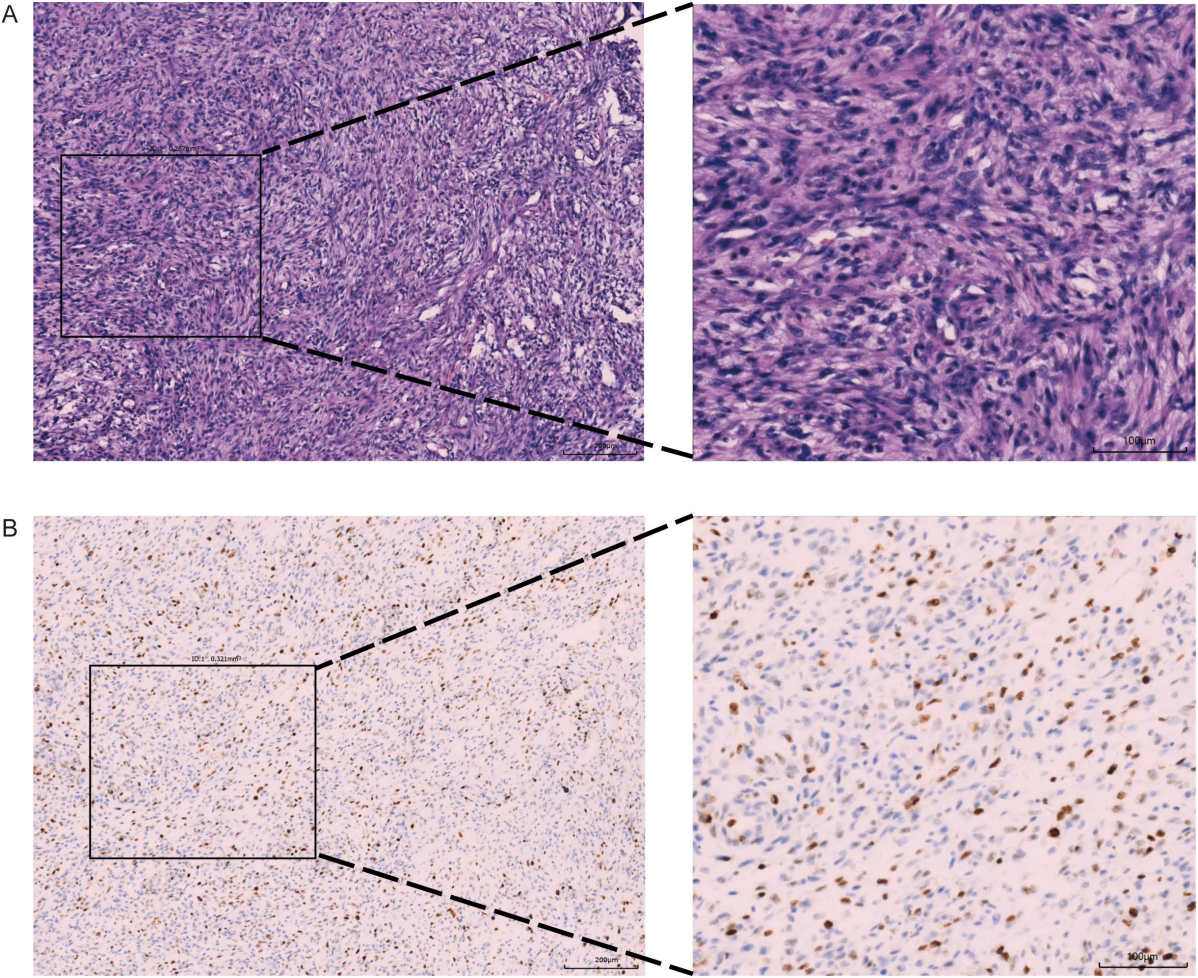
Pathological Examination Report (10X and 20X magnification): **(A)** Hematoxyin and eosin(H&E) Staining; **(B)** Ki-67 Immunohistochemistry.

On September 2, 2023, after further examinations, an emergency thoracotomy was performed. During the surgery, it was found that the tumor had filled the upper and middle sections of the left lung, compressing the lower lobe and adhering to the chest wall. The left thoracic mass was excised, chest adhesions were loosened, and part of the left rib was removed. The postoperative mass measured 12.5×3.5×3.3 cm, with a gray-white and gray-yellow appearance and firm texture (**Figure 1C**). Pathological examination indicated a phyllodes tumor with sarcomatous elements, consistent with breast origin. Immunohistochemistry showed intramembranous SMA, Caldesmon, focal Desmin, and β-catenin positivity, with a Ki-67 hotspot area of approximately 40% (**Figure 2**). Histological sections revealed a sarcoma rich in blood vessels, with foam cells, multinucleated giant cell reactions, extensive necrosis, calcification, and ossification. Postoperatively, the patient’s symptoms of chest tightness significantly improved. Given the patient’s physical condition, follow-up and further treatment were recommended after three months. During this period, genetic testing of the patient’s tissue sections and blood samples revealed a KDM6A gene mutation (c.3510dup, p.Leu1171Serfs*32, Exon 24; Geno-Truth Dx Lab). The two gene mutations and their locations are shown in **Supplementary Figure S1B**.

On December 20, 2023, during follow-up, tumor recurrence was observed, with the mass reaching nearly 8 cm (**Figure 1B**), and the recurrence was rapid, although the patient had no obvious symptoms. We then conducted a multidisciplinary team (MDT) discussion regarding the patient’s treatment plan, inviting specialists from oncology, radiation oncology, and interventional radiology. The expert consensus was that tumor embolization posed a high risk of chest bleeding, and the recommendation was to proceed with systemic chemotherapy combined with targeted therapy based on genetic testing. Therefore, we provided the NCCN-recommended first-line chemotherapy regimen, or the use of Pembrolizumab or KDM6A-related targeted therapies. However, the most common and severe side effects of Doxorubicin are myelosuppression and cardiotoxicity, and the cost of targeted therapies is 2-3 times higher than that of immunotherapy, with the local government not covering the cost of targeted drugs. Considering the side effects and financial burden, the patient and their family decided to forgo chemotherapy and targeted therapy. However, we did not give up on this patient. Upon reviewing the indications for Tislelizumab, a drug commonly used in lung cancer immunotherapy, we found that it meets the condition for “patients with other advanced solid tumors who have progressed after prior treatments and have no satisfactory alternative treatment options,” which aligns with the patient’s current condition. Additionally, the local government’s medical insurance covers the cost of immunotherapy. Therefore, we presented this treatment option to the patient and their family, and they ultimately accepted this regimen. The reason for not testing for PD-L1 expression is that before surgery, the patient had no intention of refusing postoperative adjuvant chemotherapy, so PD-L1 immunohistochemical testing was not included.

After discussion with the patient and her family, the patient decided to undergo six cycles of tislelizumab monotherapy (200 mg every 3 weeks). From January 2024 to June 2024, six treatments were completed, during which the only adverse reaction experienced by the patient was constipation. A follow-up enhanced CT report in June 2024 showed that the tumor had grown to approximately 9.1 cm, and a bone scan suggested local rib metastasis (with rib pain). Dissatisfied with the outcome, the patient and her family decided to discontinue treatment after completing the six cycles. In the subsequent follow-up, it was learned that the patient passed away on July 30, 2024, due to respiratory failure.

## Discussion

The following are the highlights of this case. First, the patient underwent a complete resection of a large-scale PT. However, only three months after surgery, the tumor recurred, and its growth rate exceeded expectations, almost returning to its original size. Secondly, we identified rare mutations in both the previous *in situ* PT and the current metastatic MPT using next-generation sequencing (NGS), respectively FGFR2 and KDM6A mutations. Due to the patient’s inability to afford targeted therapy and the side effects of systemic chemotherapy, the domestic drug tislelizumab was ultimately chosen for treatment. Tislelizumab is indicated for advanced solid tumors, and it is covered by local government health insurance. The patient received six cycles of immunotherapy, with PFS of 3.5 months. To date, there have been no studies reporting the use of tislelizumab in the treatment of metastatic MPT, and this case provides new reference and hope for PD-L1 immune checkpoint inhibitors in the treatment of advanced MPT.

The diagnosis of breast PT is challenging. On ultrasound, benign PT typically presents with characteristics such as filled gaps, no microcalcifications, leaf-like splitting, and heterogeneous internal echo patterns (6). MPT is characterized by a large, irregular mass on ultrasound. For metastatic MPT, CT is selected as the primary imaging modality, with contrast-enhanced CT providing detailed information about the tumor’s relationship with surrounding tissues and organs, as well as its blood supply. CT-guided biopsy can confirm the pathological diagnosis, but due to the tumor’s rich blood supply, there is a risk of bleeding, requiring caution during the procedure (11). On MRI, PT usually appears as a well-defined, oval-shaped mass with isosignal intensity on T1-weighted images and heterogeneous high signal intensity on T2-weighted images. MPT may exhibit irregular cystic wall changes, and on T2-weighted images, the signal intensity is lower than that of normal glandular tissue. MRI is also important for evaluating distant metastasis of MPT to the spine and brain (12). Fine needle aspiration (FNA) is difficult to distinguish breast fibroadenomas from PT, and postoperative biopsy is often relied upon. Histologically, benign phyllodes tumors display abundant intercellular material, spindle cells, and low mitotic rates (less than 5 mitoses per 10 high-power fields). Borderline phyllodes tumors exhibit some but not all malignant features. Malignant phyllodes tumors are defined by significant nuclear pleomorphism in stromal cells, excessive growth of stromal components (without epithelial components in high-power fields), diffuse stromal infiltration, increased mitotic activity (greater than 10 mitoses per 10 high-power fields), and invasive borders (13). Immunohistochemically, phyllodes tumors express p53, Ki-67, CD117, EGFR, p16, and VEGF, with the lowest positivity rates in benign tumors and the highest in malignant ones (6). When phyllodes tumors metastasize to the chest, imaging may resemble isolated fibroadenomas, while cytopathology typically reveals spindle cell soft tissue tumors. Therefore, a combination of immunohistochemistry and patient history is crucial for accurate diagnosis. The incidence of distant metastasis in malignant phyllodes tumors can reach 10%, with almost all organs potentially affected, particularly the lungs and bones (14, 15). A large cohort study found that the metastasis rates for benign, borderline, and malignant phyllodes tumors were 0%, less than 2%, and 16%, respectively (16, 17). The risk of metastasis can be assessed based on factors such as necrosis, tumor size greater than 7 cm, invasive borders, significant stromal cell density, marked stromal overgrowth, and more than 5 mitoses per 10 high-power fields (18, 19).

Benign PT and fibroadenomas have overlapping histological features, making the diagnosis challenging. PT typically contains more cellular stroma and leaf-like structures, and when differentiation is unclear, it is best diagnosed as fibroadenoma to avoid overtreatment of the patient (6). For MPT, misdiagnosis as primary sarcoma can also occur, and more often, immunohistochemistry is used to assess the degree of epithelial differentiation (6, 7).

A large study on the genomic sequencing of advanced or metastatic MPT suggests that in 135 MPT patients (20), the most common gene mutation with a frequency ≥5% is the TERT promoter mutation (69.7%). Two different assays revealed that 36.8% of the samples were PD-L1 positive (≥1% immune cells) and 21.4% were PD-L1 positive (TPS ≥1). Interestingly, one patient exhibited an FGFR3-TACC3 fusion. This study highlights the significant potential for immunotherapy or targeted therapy for MPT.

The tumor gene mutation in this patient was identified through NGS sequencing. NGS can sequence large amounts of nucleotides within a short period and at a cost affordable to individual patients (21). Compared to other biomarker detection techniques such as fluorescence *in situ* hybridization (FISH), PCR, CRISPR, and immunohistochemistry (IHC), high-throughput NGS sequencing can detect more genes and unknown mutations, making it more suitable for developing targeted drug and immunotherapy regimens for advanced or metastatic cancer patients (22). In the ESMO report on the use of NGS in advanced solid tumors in Europe (21), experts pointed out that in countries offering “tumor-agnostic targeted therapies” and in patients with advanced non-squamous NSCLC, prostate cancer, ovarian cancer, and cholangiocarcinoma, multi-gene NGS testing should be routinely conducted. Although MPT is not specifically included in this ESMO report, it is clearly stated that NGS is an important tool for identifying histological subtypes of soft tissue sarcomas, particularly in metastatic soft tissue sarcomas, making the use of NGS more reasonable. However, not all situations require NGS; for instance, in cases of NTRK mutations, where TRK inhibitors have shown efficacy, other more cost-effective testing methods are recommended as alternatives to NGS.

Fibroblast growth factor receptors (FGFRs) have increasingly been recognized as important therapeutic targets in patients with advanced refractory tumors (23), with approximately 7% of tumors carrying FGFR mutations. FGFR signaling regulates cell differentiation and proliferation, while also promoting anti-apoptotic pathways that contribute to chemotherapy resistance. FGFR mutations are most commonly found in urothelial carcinoma. Futibatinib, a next-generation irreversible FGFR1-4 inhibitor, has shown antitumor effects in FGFR-mutant tumors, particularly in cholangiocarcinoma. Goyal reported a 42% response rate to futibatinib in cholangiocarcinoma patients, with one case achieving complete remission (24). In a clinical trial (NCT02052778) involving futibatinib treatment for advanced solid tumors with FGFR2 mutations (25), the overall response rate (ORR) was 44.1%, with a median progression-free survival (mPFS) of 9.0 months. Although there is currently no study proving the efficacy of this targeted drug for MPT, we believe futibatinib holds significant potential for treating MPT or metastatic MPT with FGFR2 mutations.

KDM6A is an X-linked histone lysine demethylase, and its precise role remains unclear. A few patients with Kabuki syndrome also exhibit KDM6A mutations, leading to abnormal facial features, skeletal malformations, and cardiac and cognitive impairments. KDM6A mutations are more common in bladder cancer and breast cancer, with some evidence suggesting that KDM6A loss may promote tumor progression through the TGF-β pathway (26). Currently, no effective targeted drugs for KDM6A have been thoroughly researched. To date, fewer than 30 KDM6A inhibitors have shown limited efficacy and selectivity *in vitro* and in cell models, with most not yet entering clinical trials (27). Specific inhibitors such as GSK-J1 and GSK-J4 have been developed to target the KDM6 family. Dr. Cregan reported that preliminary testing of GSK-J1 on NCI-H226 cell proliferation showed that it could inhibit the growth of malignant pleural mesothelioma (MPM) cells with KDM6A mutations (28).

PT is relatively rare, accounting for about 0.3%-1% of all breast tumors. Of these, 10%-20% are malignant, and 9%-27% experience distant metastasis (29). The lungs are the most common site of metastasis (30, 31), although there are rare cases involving the pleura and heart (32, 33). The prognosis for metastatic malignant phyllodes tumors is generally poor, with a median survival of 5 to 30 months. Due to the rarity of distant metastasis in PT, there is currently no standardized treatment protocol for managing postoperative metastasis in PT. The NCCN guidelines recommend treating distant metastasis of phyllodes tumors according to sarcoma treatment protocols (34). Currently, there is no evidence to suggest that radiotherapy provides significant long-term survival benefits for patients with MPT or metastatic MPT. According to the 2022 NCCN Soft Tissue Sarcoma Guidelines (35), the treatment for postoperative metastasis of breast tumors should resemble that for sarcomas. Recommended chemotherapy regimens include doxorubicin, ifosfamide, and mesna, or ifosfamide, epirubicin, and mesna. Previous reports suggest that adjuvant chemotherapy has not improved metastasis-free survival and both drugs can cause bone marrow suppression, and long-term use or high doses may lead to cardiotoxicity (6). A study by Moon et al. (36) indicated that despite the lack of standardized treatment protocols for PT, adjuvant chemotherapy with doxorubicin and ifosfamide could effectively achieve complete remission of pulmonary metastasis. For large tumors, surgical resection may be necessary to alleviate symptoms and prevent complications, as in this case. To ensure complete tumor removal and reduce the risk of tumor cell spread, a thoracotomy was performed. During this procedure, part of the fourth rib, the upper lobe of the left lung, and the pericardium were excised. The tumor capsule was intact, and the surgery was completed with minimal bleeding and no major complications.

Traditionally, immune checkpoint inhibitors (ICIs) are used in combination with chemotherapy, not only converting “cold” tumors to “hot” tumors but also reducing suppressive immune cells, enhancing tumor antigen presentation, and thereby improving tumor-killing activity (37, 38). Therefore, the combined application of immunotherapy and chemotherapy holds promise for future treatments of metastatic MPT. According to Pollack et al. (39), the combination of doxorubicin and pembrolizumab resulted in a median PFS of 8.1 months and a significantly higher median OS of 27.6 months. This study fills a gap in the treatment of breast phyllodes tumor metastasis to the chest and continues to explore the combination of Tislelizumab with various chemotherapy agents as a new direction for treating distant metastasis in MPT.

## Conclusion

Given the potential for tumor recurrence following complete surgical excision, it remains essential to monitor patients consistently. The rarity of PT metastasizing to the lungs enhances our comprehension of their progression, pathology, and imaging characteristics, thereby enriching our treatment strategies. Furthermore, the limited availability of KDM6A-targeted therapies underscores the necessity for ongoing research in this area.

## Data Availability

The datasets presented in this study can be found in online repositories. The names of the repository/repositories and accession number(s) can be found in the article/**Supplementary Material**.

## Supplementary material

Supplementary Figure 1
**(A)** Histological sections of previous PT; **(B)** The location of mutated genes on the chromosomes.
