# Supplementary figures and images for: Unexpected outcomes of tislelizumab treatment in thoracic metastasis of malignant phyllodes tumors: a case report and literature review
Source: Front Oncol. 2025 Apr 14;15:1535653. doi: 10.3389/fonc.2025.1535653 (PMC12034558; doi:10.3389/fonc.2025.1535653)

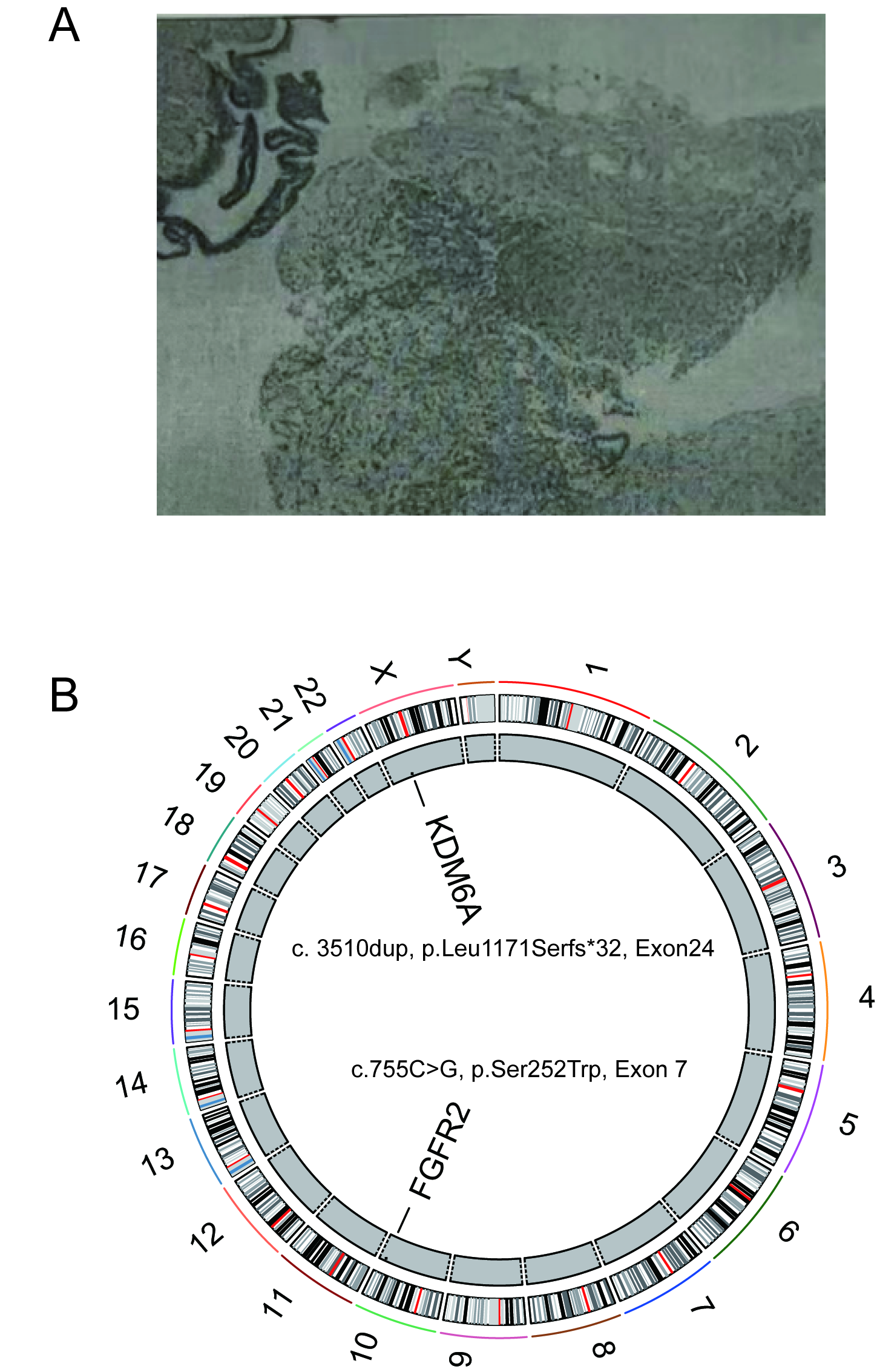

Supplement: Supplementary Figure 1 — (A) Histological sections of previous PT; (B) The location of mutated genes on the chromosomes. [file Image1.tif]
